# Draft Genome Sequence of the *Suttonella* *ornithocola* Bacterium

**DOI:** 10.1128/genomeA.01592-16

**Published:** 2017-02-16

**Authors:** Hiba Waldman Ben-Asher, Rebecca Yerushalmi, Chaim Wachtel, Efrat Barbiro-Michaely, David Sompolinsky, Doron Gerber

**Affiliations:** a Everard and Mina Goodman Faculty of Life Sciences, Bar-Ilan University, Ramat Gan, Israel; b Laboratory of Microbiology, Mayanei Hayeshua Medical Center, Bnei Brak, Israel

## Abstract

We report here the draft genome sequence of the *Suttonella ornithocola* bacterium. To date, this bacterium, found in birds, passed only phylogenetic and phenotypic analyses. To our knowledge, this is the first publication of the *Suttonella ornithocola* genome sequence. The genetic profile provides a basis for further analysis of its infection pathways.

## GENOME ANNOUNCEMENT

A variety of infectious diseases have been reported to cause morbidity and mortality of garden birds in Great Britain and continental Europe. The *Suttonella ornithocola* bacterium was first isolated from the lungs of a British tit species in 1996. This bacterium was defined as a novel species belonging to the *Cardiobacteriaceae* family (1). In 2011, the *Suttonella ornithocola* bacterium was associated with infection in tits, causing acute necrotizing pneumonitis (Paridae) (2). Relatively little is known about *Suttonella ornithocola* in British tit species or other species.

The *Suttonella ornithocola* bacterium was collected from the culture collection University of Göteborg (CCUG). *De novo* sequencing of *Suttonella ornithocola* was conducted by the Bar-Ilan University Microarray Unit, Israel. DNA libraries were generated as follows: DNA was extracted from 2 ml of culture using the DNeasy blood and tissue kit (Qiagen), as per the manufacturer’s instructions. For each sample, 100 μg of DNA was used to prepare a barcoded library for sequencing, using the Ion Xpress Plus fragment library kit (Life Technologies, Inc.). Briefly, DNA was fragmented with Ion Shear enzyme mix II, and adaptors and barcodes were ligated to the fragmented DNA. The ligated DNA was then size-selected on a 2% E-Gel SizeSelect agarose gel (Invitrogen). The size-selected library was then amplified using the primers supplied in the Ion Xpress Plus fragment library kit (Life Technologies, Inc.). The concentrations of the libraries were measured using a Qubit double-stranded DNA (dsDNA) high-sensitivity (HS) assay kit, and the size distribution was analyzed with an Agilent high-sensitivity DNA kit. Libraries were prepared for sequencing on a One Touch 2 using the Ion PGM Template OT2 200 kit (Life Technologies, Inc.). The sequencing was then performed on an Ion Torrent PGM using Ion PGM sequencing 200 kit version 2 (Life Technologies, Inc.).

An overall 1.6 million reads (1,630,659 reads) with a mean length of 187 bp were received. Data were analyzed using the *de novo* MIRA assembler (version 3.4.2.0) (3). The final draft comprises 327 contigs (longer than 500 bp), with a mean size of 14,389 bp and a maximum length of 51,440 bp. Sequencing revealed that the total length of the genome was 2,519,682 bp, with a mean GC content of 39% and an approximate 121-fold coverage of the genome. Contig annotation was conducted using the Rapid Annotations using Subsystems Technology (RAST) server (4).

The final draft contains 2,610 coding sequences (of which 1,685 coding sequences possess annotated functions and 925 are hypothetical proteins) and 53 RNA genes.

### Accession number(s).

This whole-genome shotgun project has been deposited at DDBJ/EMBL/GenBank under the accession number LWHB00000000. The version described in this paper is version LWHB01000000.
